# The role of the early video-assisted thoracoscopic surgery in children with pleural empyema

**DOI:** 10.1007/s00383-024-05715-y

**Published:** 2024-05-18

**Authors:** Marco Di Mitri, Eduje Thomas, Eleonora Capano, Cristian Bisanti, Simone D’Antonio, Michele Libri, Tommaso Gargano, Mario Lima

**Affiliations:** Pediatric Surgery Department, IRCCS Sant’Orsola-Malpighi Polyclinic, Alma Mater Studiorum—University of Bologna, 40126 Bologna, Italy

**Keywords:** Pleural empyema, Thoracoscopy, VATS, Video assisted thoracoscopic surgery, Minimally invasive surgery, Children

## Abstract

**Purpose:**

Pleural empyema (PE) is a collection of purulent material in the pleural space. PE’s management in children is a challenge and an inappropriate diagnostic-therapeutic work up can lead to serious short and long-term complications. The aim of this study is to define the correct timing to approach a pediatric PE by video-assisted thoracoscopic surgery (VATS).

**Methods:**

A retrospective observational study was conducted including pediatric patients who underwent video-assisted thoracoscopy for pleural empyema between May 2005 and September 2022.

**Results:**

62 patients were subjected to VATS for PE (32 in Group Early VATS, 30 in Group Late VATS). It emerged that the elapsed period between the onset of symptoms and surgery correlates in a statistically significant way with the post-operative stay in intensive care (z score 4.3 and p value < 0.0001) and the analysis between early VATS, late VATS and postoperative hospitalization showed a statistically significant reduction of the post-operative hospitalization in the early VATS groups (p value < 0.02).

**Conclusions:**

VATS resulted to be safe and effective for the treatment of PE in children, and an early minimally invasive thoracoscopic intervention (early VATS) correlates with better outcomes, specifically in terms of intensive care hospitalization and overall hospitalization.

## Background

Pleural empyema (PE) is defined as a collection of purulent material in the pleural space. PE has a multifactorial etiology, with infections being the leading cause [1]. Other causes are thoracic surgery, trauma, and esophageal perforation [2].

In Europe, PE incidence has increased in the last year reaching around 3 cases per 100.000 children/year [3–5]. Moreover, if we consider that incidence of community-acquired pneumonia (CAP) has decreased, especially after the introduction of the anti-Pneumococcal vaccine, on the other hand, the rate of complications associated with pneumonia has risen.

**Fig. 1:**
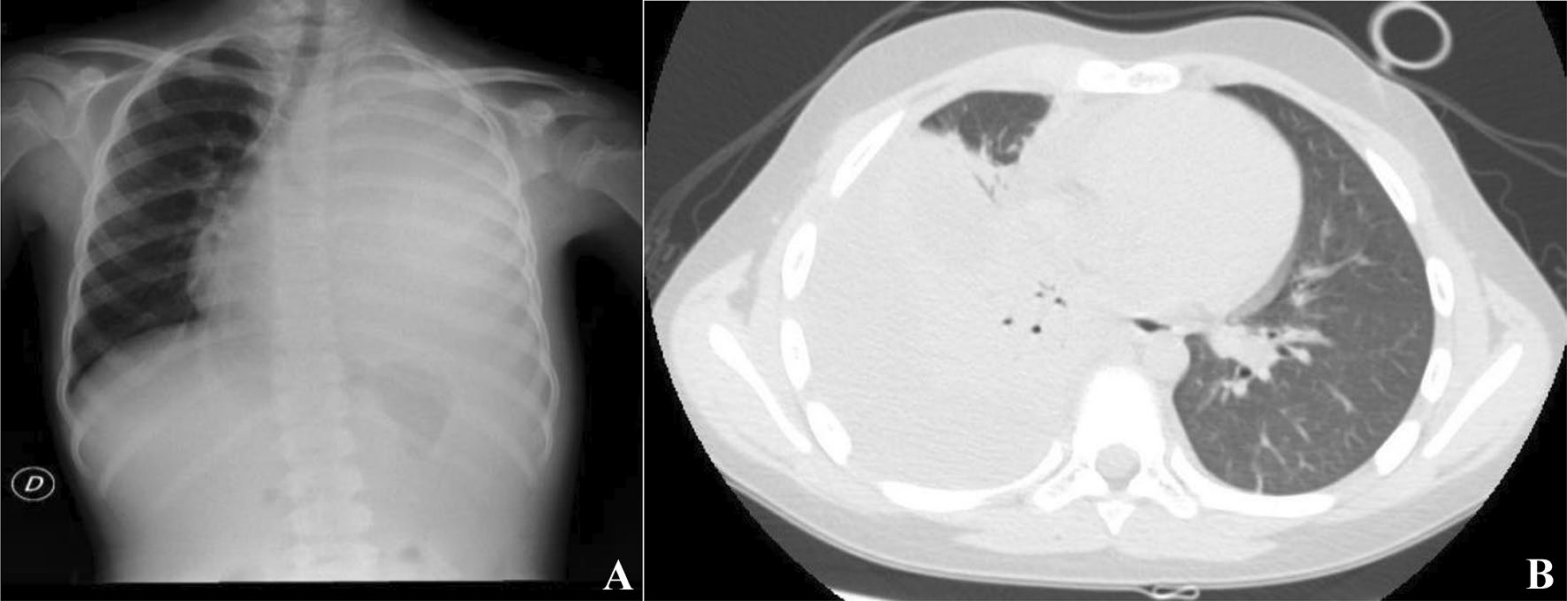
**a** Chest XR of left pleural empyema. **b** CT scan of right pleural empyema

The reasons of this increasing trend are still being studied, but among the analyzed and potentially involved factors, the most important seems to be new bacterial resistances and virulence, which are linked to the antibiotic prescription habits. [5] From a microbiological point of view, the most frequently isolated pathogen is Streptococcus Pneumoniae, followed by Streptococcus Pyogenes and Staphylococcus Aureus [6, 7]. These bacteria reach the pleural space mainly by contiguity, through the blood stream or via the lymphatic route. Clinically, PE manifests itself, in most cases, with the typical pneumonic symptoms, meaning cough, dyspnea, fever, asthenia, exercise intolerance, and pleuritic chest pain [1, 8]. Physical examination may reveal impaired unilateral chest expansion capacity, dullness on percussion, and reduced or absent vesicular murmur [9]. In addition to the medical history and clinical signs, instrumental investigations are vital for the diagnosis of PE. Chest X-ray (XR) is the first-level examination. In the past years, pulmonary ultrasound has demonstrated the same sensibility and specificity of XR, playing a key role in the follow-up, as it yields a more accurate evaluation of the pleural effusion, and allows to discern a simple liquid effusion from a purulent effusion with or without the presence of septa. Chest CT scan is a second level investigation which might represent a crucial exam before surgically approaching a patient with pleural empyema, because of its high resolution of anatomical details, not only on the quality of the effusions but also on the pulmonary parenchyma. [10, 11] (Fig. 1a-b).

**Fig. 2:**
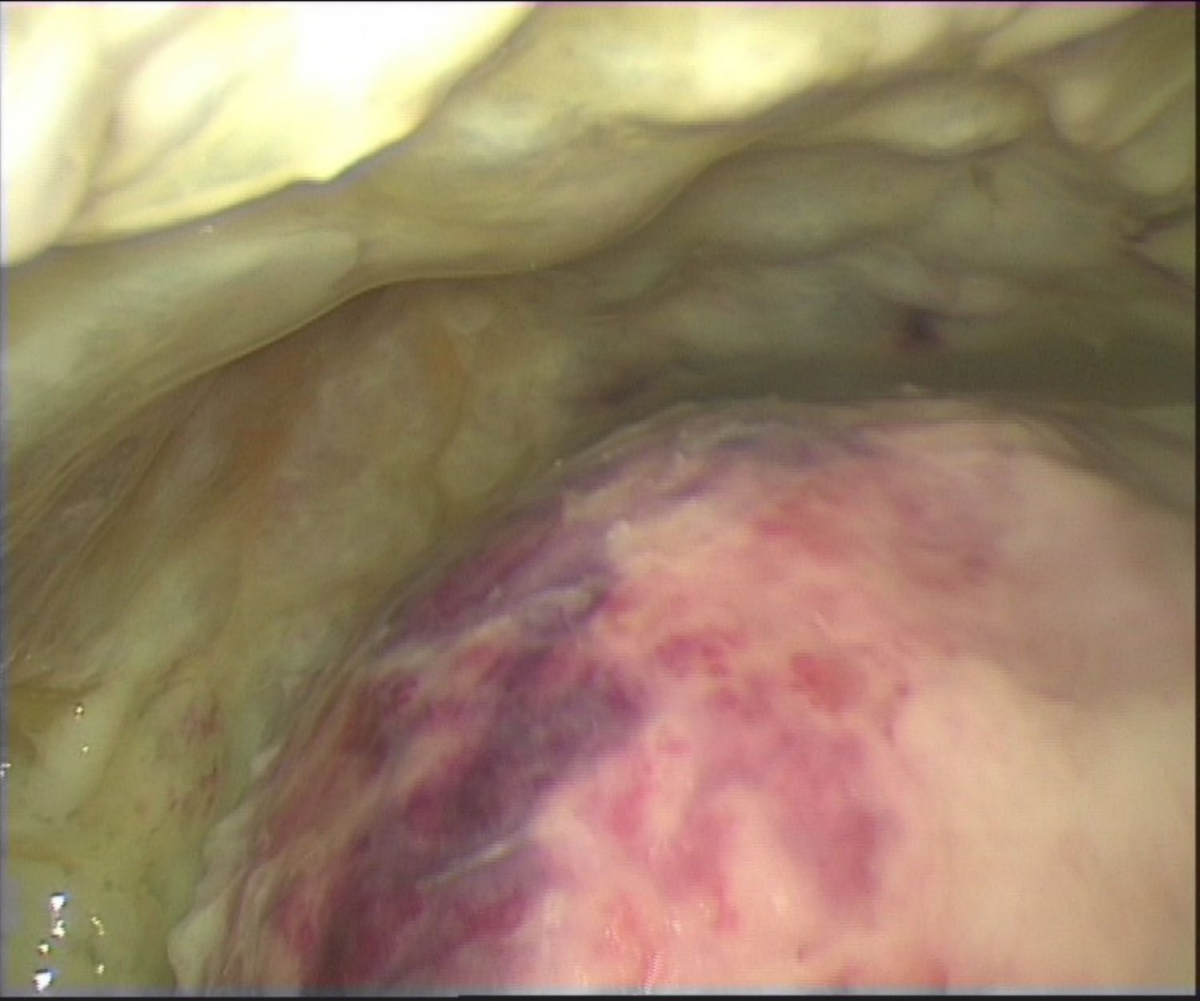
Thoracoscopic view of stage II of pleural empyema

Progression to the fibrinopurulent stage (II) can occur within a few hours if an effective treatment is not implemented. This stage is characterized by the deposition of fibrin membranes in the pleural space, with consequent formation of weirs, compartmentalization, and the development of loculations and isolated fluid collections. (Fig. 2).

**Fig. 3:**
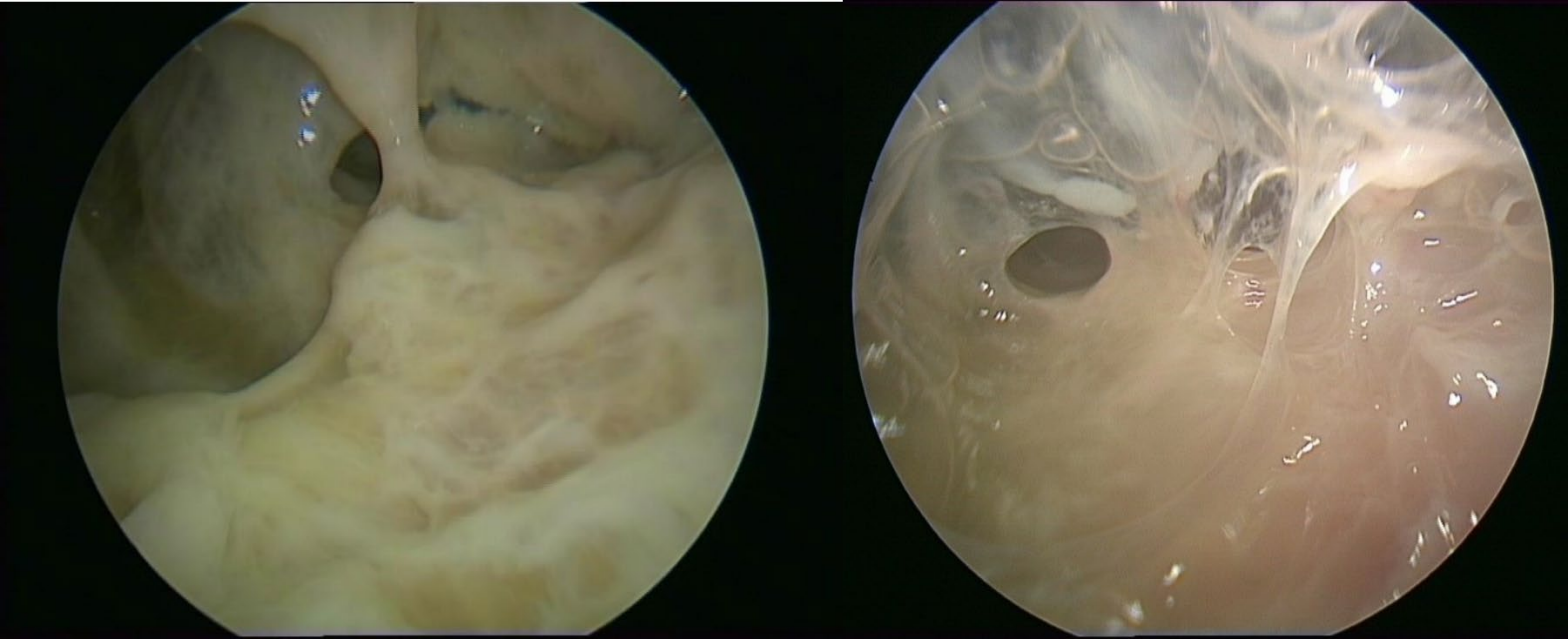
Stage 3: Thoracoscopic view of pleural empyema with extensive septa

The third and final stage (III) (Fig. 3), defined as organized, is triggered by the proliferation of fibroblasts and their subsequent invasion of the pleural space, starting from both the parietal and visceral pleura. In this stage, fibroblasts transform the fibrin membranes into a thick and inelastic layer, which can significantly compromise lung function, limiting re-expansion and leading to a state called "trapped lung" [12, 13].

The management of pediatric PE is a challenge for pediatricians, pediatric surgeons, and anesthesiologists. Different therapeutic approaches have been described in literature, including antibiotic therapy (AT), which may be associated with evacuative thoracentesis, placement of chest drainage (CD), with the possibility of intra-pleural administration of fibrinolytic drugs, and surgery.

Surgery entails different approaches such as video-assisted thoracoscopic surgery (VATS) or thoracotomy. [14] However, to date, a consensus on the most appropriate treatment of PE has not been reached.

Previously, AT was adopted as a first-line approach, followed by chest tube placement only if unsuccessful. In the last years, due to the progress in the field of minimally invasive surgery, resulting in less recovery time, shorter hospitalization, less post-operative pain and better aesthetic results, surgery was reconsidered as a possible therapeutic approach in earlier stages [15–18].

The safety and the efficacy of VATS, pleural drainage or evacuation have been established by several studies, while the timing of the surgical procedure remains controversial [13, 17–20].

The aim of this study is to define the correct timing to approach a pediatric PE by VATS.

## Methods

A retrospective observational study was conducted at our Department of Pediatric Surgery, IRCCS Sant’Orsola-Malpighi, Alma Mater Studiorum, University Hospital of Bologna. All patients involved in the study signed the consent to the publication and processing of the data.

We enrolled all the patients who subjected to VATS for PE between May 2005 and September 2022, and we divided them in two groups: early VATS, who underwent surgery within 5 days from admission to the emergency department, and late VATS, who underwent surgery after 5 days from admission, according to the results highlighted by Pappalardo et al., [36].

We analyzed the efficacy of the early minimally invasive thoracoscopic treatment, in comparison to a conservative management and late VATS.

We collected data from medical records and analyzed data regarding:

- Personal data.
- PE onset (onset symptoms, time from symptoms to emergency area access).
- Clinical conditions at hospital admission.
- Hospitalization.

### Statistical analysis

Data are reported as population frequency (%) and mean with standard deviation (mean ± SD). For a more accurate estimate of the central tendency, in case of asymmetric distribution and mean values that deviate from the median, the median value and interquartile range are also reported.

Quantitative variables were analyzed with student’s t test or Mann–Whitney U test in case of deviation from normality assessed with Shapiro–Wilk test. A p value below 0,05 was considered statistically significant.

## Surgical procedure

Surgery was performed by thoracoscopic approach in lateral decubitus, on the opposite side to localization of the empyema. The first 5 mm trocar is usually placed anterior to the midaxillary line approximately at the level of the fifth intercostal space, by open technique.

**Fig. 4:**
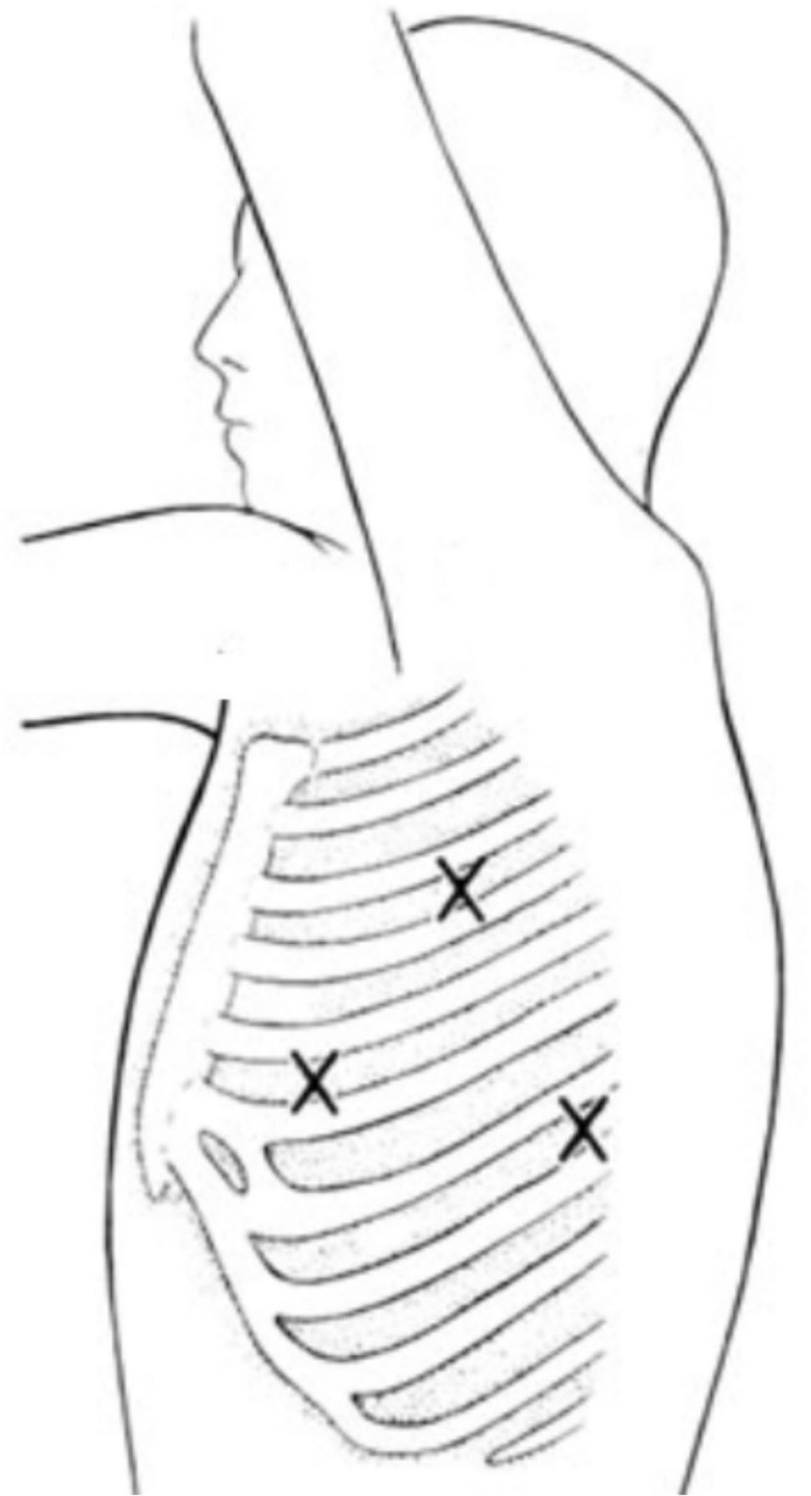
Trocars position

A pneumothorax with low CO_2_ flow and a pressure of 5–6 mmHg can be created after checking the correct positioning of the trocars. After creating the necessary space, two 5 mm trocars are placed. Figure 4

First, we aspirate the pleural effusions, which will be sent for laboratory tests. Second, forceps are introduced to remove all fibrin septa from the pleural surface. After decortication, we clean the thoracic cavity with saline solution 0,9%. The procedure ends with the positioning of two chest tubes in the posteroinferior and anterosuperior position using the trocars entry site.

## Results

### Population

We enrolled 66 patients who underwent a surgical treatment for PE, of whom 40.9% (n = 27) were female, and 59.1% (n = 39) were male. We excluded 4 patients treated with open surgery.

The median age at diagnosis was 5 years for male patients and 6 years for female patients (range 5 months—23 years). In particular, 68.2% of the total cases (n = 45) of pleural empyema were diagnosed within 5 years of age, while 31.8% of cases (n = 21) above 6 years. (Table 1) Chest tubes are placed only after the thoracoscopic exploration and not as a first line treatment for pleural empyema. Their purpose is to prevent postoperative recurrence of pleural effusions and monitor any potential pneumothorax.

**Table 1:** Patients characteristics

Population	
Sex	M: 39; F = 27
Age (years)	M: 5; F = 6 (range 5 month–23 years)
Symptoms	
Cough	65.2%
Fever	100%
Dyspnea	36.4%

A chest tube was placed in all patients who underwent late VATS, while in only 22% of patients underwent early VATS.

## Onset symptoms

At onset, 65.2% (n = 43) of patients presented cough and, on average, the symptom had been present for 8 days at the time of admission (SD ± 10, median = 5, IQ = 2;10). Fever was present in all patients at the time of admission for an average of 6 days (SD ± 8, median = 4, IQ = 3;7), with a mean maximum registered temperature of 39.4 °C (SD ± 0.21).

Dyspnea was present in only 36,4% (n = 24) of patients with a mean of 4 days (SD ± 3) in 36.4% while the chest pain was experienced in 30.3% (n = 20) of cases, for a mean of 4 days (SD ± 3). All patients who came from home underwent antibiotic therapy with amoxicillin + clavulanic acid, immediately switched to a third-generation cephalosporin.

Patients transferred from other departments were already on a third-generation cephalosporin. Despite this, no statistically significant differences were shown between the two groups.

## Clinical conditions

At admission, the mean peripheral oxygen saturation (SpO2) was 95.48% (SD ± 3), and 18.2% (n = 12) of patients required respiratory support; in particular, 6.1% (n = 4) were supported with nasal goggles, 3% (n = 2) by Ventimask, 4.5% (n = 3) with HFNC (High Flow Nasal Cannula), and 4.5% (n = 3) underwent mechanical ventilation.

The assessment of vital signs conducted in the emergency area recorded a mean heart rate of 137 beats/minute (SD ± 18), a mean Systolic Blood Pressure of 106 mmHg (SD ± 13), and a mean Diastolic Blood Pressure of 63 mmHg (SD ± 15).

The blood gas values showed mean blood pH value of 7.40 (SD ± 0.7), a mean P/F ratio of 260 (SD ± 92) and a mean lactate value of 0.86 (SD ± 0.27).

## Surgery

The 93.9% (n = 62) underwent thoracoscopic evacuation and toileting of the pleural space using VATS, while 6.1% (n = 4) underwent surgery with open thoracotomy approach for several complications (necrotizing pneumonia).

All patients included in the study were subjected to chest X-ray and a pulmonary ultrasound. A preoperative CT scan was then performed prior to surgery and the radiological investigations revealed mediastinal shift, pleural septations, and extensive pleural effusions. We divided patients subjected to VATS in two groups: we collected 32 patients in the early VATS group (surgery within 5 days from hospitalization) and 30 patients in the late VATS group (surgery after 5 days from hospitalization). (Table 2).

**Table 2:**
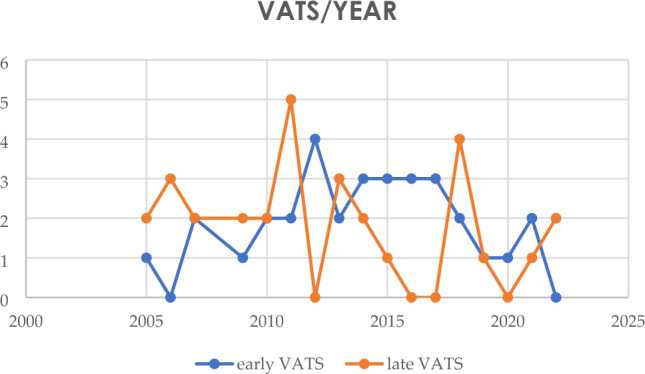
Rate of early vs late VATS/year

Furthermore, the time between symptoms onset and the execution of the surgery was 14 days on average (SD ± 10).

The mean duration of the thoracoscopic procedure was 109 min (SD ± 37), and in all cases we placed 2 chest tubes, at the apex and at the base of the pleural cavity.

No intraoperative complications were reported, and the intraoperative conversion rate to open procedure was 3.2% (n = 2).

Lung parenchyma resection was required in 3,2% (n = 2) of cases and was performed through thoracotomy. Drainages were removed based on clinical conditions on VII (DS ± 4) post-operative day (POD) for the apical drainage and IX POD (DS ± 4) for the basal drainage.

## Hospitalization

The hospitalization in Pediatric Intensive Care Unit was necessary in all cases following surgery with a mean duration of 7 days (SD ± 5, IQ = 3;6) for early VATS group and 12 days (SD ± 6, IQ = 3;13) for late VATS group. The overall hospitalization duration was of 22 days (SD ± 13, IQ = 13;27) for early VATS group and 30 days (SD ± 10, IQ = 20;38) for late VATS group.

Statistical analysis of the collected data showed a statistically significant correlation between the duration of symptoms before arrival in the emergency department and the length of hospital stay (z-score—8.7, p value < 0.01).

Furthermore, it emerged that the elapsed period between the onset of symptoms and surgery correlates in a statistically significant way with the post-operative stay in intensive care (z score 4.3 and p value < 0.0001). In addition, the correlation between early VATS, late VATS and postoperative hospitalization showed a statistically significant reduction in post-operative hospitalization in the early VATS group (p value < 0.02).

## Discussion

Pleural empyema represents one of the main complications of pneumonia in pediatric patients, with an incidence that is increasing year by year. PE requires prolonged periods of hospitalization and can compromise the respiratory function of the patient.

Despite its relevant epidemiology, the management of pediatric PE has not reached a consensus, and, in clinical practice, the therapeutic approach varies within different centers [1, 3, 5, 8].

The lack of consensus is due to poor the pediatric literature available and to the discrepancy of the results reported by existing studies.

However, we must consider that the mortality rate among pediatric patients is negligible if compared with the adult population. Therefore, regardless of the type of approach adopted, most patients achieve full healing and resolution.

Nevertheless, clinical practice must be aimed at guaranteeing the most adequate and effective therapeutic management, defined by a better prognosis, fewer post-operative sequelae, complete restoration of respiratory function and short hospitalization.

Manoharan et al. investigated which is the best way to manage pediatric PE, concluding that only CD is associated with a significant failure rate due to its inability to clear loculated effusion. On the other hand, VATS and the intrapleural fibrinolytic therapy proved to be equally effective and safe, considering as candidates for decortication children who presented in the final stage [21].

A moderate consensus reported in literature advocated a conservative management for the initial stage of pleural empyema. According to the British Thoracic Society guidelines, pleural effusion with impaired respiratory function can be managed by drainage and antibiotics. The most controversial aspect of the treatment of pediatric pleural empyema, concerns the choice between a conservative approach, based on antibiotic therapy, chest drainage, intra-pleural fibrinolysis, and a surgical approach, by VATS.

## Conservative management

The conservative management is based on antibiotic therapy and several studies support its effectiveness. Lohuis et al. reported the results of a study conducted on a large population of 136 patients, aged 1–18 years. They treated 117 (87%) patients by antibiotic therapy and in 19 (14%) patients, they placed a chest tube after the unsuccess of AT. They correlated the outcomes with presence of mediastinal shift, pleural septations/pockets, pleural thickening, or extensive effusions, concluding that if none of these conditions are present, recovery is achieved with antibiotic therapy alone in most cases [22]. Lohuis et al., R. Epaud et al. showed that in patients with small effusions, and in the absence of mediastinal shift or respiratory distress, it is possible to reach the healing only by antibiotic therapy, reserving operative procedures for the most severe cases, without any differences in terms of short-term or long-term outcomes [23].

Leoni et al. sent a questionnaire according to the DELPHI method to French experts in the field (pediatric pulmonologists and infectious disease specialists). The responses showed that in the absence of clinical signs of severity, the prescription of an intravenous AT is consensual but there is no agreement on the choice of the drug to use, on the duration of AT and on the treatment of severe PE. [24] Meyer Sauteur et al. tried to verify the role of Amoxicillin/Clavulanate analyzing a cohort of 139 patients under 16 years old with pleural effusions/pleural empyema. They achieved a full recovery in more than 95% of children. The real limit of the study was the inclusion of patients with pleural effusions and patients with pleural empyema in the same group and the lack of a staging of the empyema [25].

The association of both AT and CD has been described as a possible therapeutic approach. Considering the chest tube placement, several studies comparing repeated thoracentesis, and placement of a permanent drain, have been conducted reporting conflicting results. Mitri et al. studied a population of 67 patients who underwent one of the two procedures, showing similar complication rates and hospital stays but they correlated thoracentesis with significantly higher re-operation rates (p < 0,05) [23]. Similarly, Shoseyov et al. compared, in a prospective study involving 67 patients, repeated thoracentesis and chest drain tube placement. In this study, no significant differences were found between the two groups, so the authors concluded that the effectiveness of the two procedures is similar [26]. Satysh et al. analyzed data related to 14 pediatric patients, treated with an association of AT and CD, achieving a complete resolution of the pathological condition in all cases, with an average hospital stay of 14 days. Despite their results, the study lacked relevant information such as clinical features, mediastinal shift, and pleural septations/pockets [27]. The retrospective study by Chan et al. considered a population of 54 patients of which the 87% were treated by CD and the 39% of patients underwent decortication for unsatisfactory response to medical treatment. They showed a statistically significant result comparing the delay of the treatment and the need for surgery, reporting how late treatment was associated with a major probability to be subjected to a surgical procedure. Therefore, they concluded that surgery should be reserved for patients with organized, multiloculated empyema with lung entrapment and early CD for the other cases. As in the previous study, the average period of hospitalization recorded was 14 days [28].

The natural pathological evolution of PE leads to the deposition of fibrin and septa formation, containing purulent material, which make transthoracic drainage alone sometimes insufficient.

For this reason, some authors described the use of intra-pleural fibrinolysis associated to CD as a therapeutic option. It consists in the instillation of fibrinolytic drugs such as Streptokinase, Urokinase and Alteplase, inside the pleural space, to obtain the lysis of septa and to restore the correct filtration and reabsorption of the pleural fluid.

Seven different studies, concerning the efficacy of fibrinolytic therapy, have highlighted how this approach has guaranteed an increase in the amount of pleural fluid drained, through chest drainage, and a favorable outcome, without the need for surgery, in 90% of patients considered (n = 136) [29–35].

Specifically, Barbato et al. compared two groups of patients with PE, the first treated with CD and fibrinolytic agents (urokinase group–n = 17), and the second treated with CD alone (historical group–n = 11). In the urokinase group, 5 patients needed surgical debridement while in the historical group surgery was necessary in 9 cases. Moreover, the main hospitalization was 17 days in the first group and 24 days in the second group, but a similar occurrence of pneumothorax was reported in both series. So, despite the limited of the sample size, they concluded that fibrinolytic therapy led to a reduction in hospital stay (P = 0.02), as well as in the need for surgery (P = 0.02) [29].

Krishnan et al. analyzed 9 patients, aged between 6 months and 6 years, who underwent intra-pleural fibrinolysis, following failure of antibiotic therapy and chest drainage, observing how 8 patients responded to treatment within 3 days, defined by the increase of the fluid drainage and reporting a resolution of the clinical symptoms. A second instillation was necessary in only one case. The authors therefore concluded that intra-pleural fibrinolysis represents an effective solution to avoid surgery and reduce hospitalization [31]. Encouraging results were reported from the study of Wells et al. They conducted a retrospective study of 71 pediatric patients undergoing fibrinolytic therapy with alteplase or urokinase, reporting a 98% success rate with the first, and 100% with the second, in the absence of major complications.

Considering results the authors affirmed the efficacy of this therapeutic approach, emphasizing a slight superiority of Alteplase (P < 0.001), compared to Urokinase (P < 0.002). [35].

## Video-assisted thoracoscopy

As previously stated, PE can be treated surgically with a thoracoscopic or thoracotomy approach.

In the past, surgery represented the second-line treatment, reserved only in case of failure of the conservative therapy.

Advances in the field of minimally invasive surgery, with the miniaturization of surgical instruments, shifted the focus of interest towards videothoracoscopy, reporting less morbidity, less post-operative pain, less scars and a shorter postoperative recovery time compared with thoracotomy.

If the advantages of thoracoscopy for PE management compared to thoracotomy are extensively described in literature, the same cannot be said for the timing of surgery.

However, in the recent decades, studies published in literature showed how early surgery can guarantee a series of benefits, such as a reduction in hospital stay, fewer complications, and a faster recovery of respiratory function. Indeed, several studies in literature demonstrated the safety and efficacy of VATS in the treatment of PE in the pediatric population, observing how this approach is associated with a short recovery time, excellent aesthetic results, reduced post-operative pain and low complication rate [19, 20].

In particular, the large series by Lamas-Pinheiro et al. considered 91 patients who underwent primary VATS between 2006 and 2014. Conversion to open approach was required in 21 cases and 6 patients required a redo due to failure to improve the clinical picture of the patient. The authors concluded defining the thoracoscopic approach for empyema not only feasible and safe, but also crucial in avoiding a significant number of thoracotomies after a short learning curve, as necrotizing pneumonia may be associated with a higher risk of reintervention [20].

Furthermore, in 2018, a systematic review and meta-analysis was published by Pacilli et al. with the aim of identifying which approach, between VATS and intra-pleural fibrinolysis, guarantees the best clinical outcome. The analysis of 10 studies failed to find significant differences in the rate of peri-operative complications (RR 0.6 [CI: 0.3–1.2], p = 0.2; I^2^ = 0.0%; p = 0.6), while it emerged that the need for re-operation (RR 0.55 [CI: 0.34–0.88], p = 0.01; I^2^ = 14.4%; p = 0.3) and the length of hospital stay (SDM −0.45 [CI: −0.78 to −0.12], p = 0.007; I^2^ = 88%; p = 0.001) were significantly lower in cases of pleural empyema treated by VATS [16].

The study conducted by Pogorelic et al. analyzed data relating to 21 pediatric patients who underwent early VATS, evaluating the outcome of an early video-thoracoscopic decortication. The different cases of empyema were divided into three evolutionary stages (I, II and III), and it was observed that the post-operative outcomes were significantly better in the first two stages, in terms of total hospital stay and intensive care stay, permanence of the chest tube, and duration of postoperative hyperpyrexia.

The authors, therefore, concluded that early VATS represents an effective and advantageous approach, to improve the healing and to reduce the hospital stays [15]. Also, Yan Le Ho divided the pediatric patients with PE into three groups based on empyema stage. Analyzing 30 patients they showed how surgical decortication represents an excellent modality for managing stage 2 and 3 pediatric empyema because it guarantees a rapid resolution of symptoms with good clinical outcomes if performed promptly. Contrary, delayed referral may result in a more protracted clinical course [38].

Concluding, despite the efficacy and safety of thoracoscopy has been showed, the most appropriate timing of intervention remains controversial.

The results of our study are in line with the latest studies reported. First, the safety and efficacy of the videothoracoscopic procedure, reported in the literature, were confirmed by the analysis of the data collected in our series: no intra-operative complications were recorded, and only 4 cases required a conversion to open thoracotomy approach to perform a lung resection for extensive necrosis of the lung parenchyma.

As previously stated, a significant correlation was found between the duration of symptoms before arrival at the emergency department and the total hospital stay; this aspect highlights the need to identify early cases of pneumonia complicated by the presence of a pleural empyema, in order to achieve a fast and adequate therapeutic work-up, not only for surgery, but also if antibiotic therapy or pleural drainage are chosen as first line treatment.

From this observation it follows that an early treatment could ensure a faster post-operative recovery, and therefore a better outcome for the patient.

This result appears to agree with the study by Pogorelic et al.: a shorter time interval between onset of symptoms and treatment translates into a lower evolutionary stage of the pathological process taking place in the pleural space, and, therefore, a faster recovery and shorter length of hospital stay.

Finally, starting from the distinction between early and late VATS based on the timeline, it was highlighted how the execution of the surgery within the first 5 days of hospitalization results in a shorter duration of the total hospitalization period. Based on literature, the early diagnosis of PE is necessary to improve the management and guarantee a rapid and full recovery. Chalmers et al. presented a score system to identify potential predictors of complicated parapneumonic effusion/empyema in patients with community acquired pneumonia by using clinical and simple laboratory variables like hemoglobin, serum C-reactive protein, serum albumin levels and total leukocyte counts. They included 269 patients were included in the study and 92 patients (7.2%) developed complicated parapneumonic effusion or empyema [12].

### Limitation of the study

However, it must be emphasized that this study has limitations, such as the retrospective nature and the absence of a comparison with patients treated by antibiotic therapy or by chest drain tube placement.

## Conclusions

In conclusion, our study investigates the role of minimally invasive surgery in management of pediatric patients with pleural empyema.

Specifically, the safety and efficacy of VATS in the treatment of PE has been demonstrated, in agreement with the data reported by the most recent publications.

Furthermore, from the statistical analysis of the collected data, it emerged that an early minimally invasive thoracoscopic intervention (early VATS) correlates with a better patient outcome, especially in terms of intensive care hospitalization and overall hospitalization.

This result appears to be interesting and relevant in defining the timing of surgery, in the context of a pathology for which there is a lack of scientific evidence to guide clinical practice.

It is therefore possible to conclude that, despite the absence of a consensus, in our experience, the early minimally invasive surgical approach represents a valid therapeutic possibility in the management of pediatric pleural empyema.

Therefore, it would be desirable to conduct multicentric and, if possible, prospective study, to strengthen the statistical significance of our data.

Furthermore, an interesting field of research, in relation to which there are few existing studies, is represented by the long-term outcome and follow-up of the pathology: in particular, it would be useful to investigate the potential impact of pleural empyema on the respiratory function of the pediatric patients.

## Data Availability

No datasets were generated or analysed during the current study.

## Abbreviations

PE: Pleural empyema
CAP: Community acquired pneumonia
XR: X-ray
AT: Antibiotic therapy
CD: Chest drainage
VATS: Video-assisted thoracoscopic surgery
POD: Post-operative day
